# Effectiveness of primary-contact physiotherapy in managing musculoskeletal conditions in emergency departments: protocol for the RESHAP-ED randomised controlled trial

**DOI:** 10.1136/bmjopen-2024-096044

**Published:** 2025-03-12

**Authors:** Tarcisio F de Campos, Danielle Coombs, Chathurani Sigera, Christopher Williams, Eileen Rogan, James Edwards, Kirsten McCaffery, Kirsten Howard, Laurent Billot, Chris Maher, Gustavo C Machado, RESHAP-ED Investigators, Rachelle Buchbinder

**Affiliations:** 1 School of Public Health, Faculty of Medicine and Health, The University of Sydney, Sydney, New South Wales, Australia; 2 Institute for Musculoskeletal Health, Sydney Local Health District, Sydney, New South Wales, Australia; 3 University Centre for Rural Health, School of Health Sciences, The University of Sydney, Lismore, New South Wales, Australia; 4 Research and Knowledge Translation Directorate, Mid North Coast Local Health District, Port Macquarie, New South Wales, Australia; 5 Emergency Department, Canterbury Hospital, Sydney Local Health District, Sydney, New South Wales, Australia; 6 Emergency Department, Royal Prince Alfred Hospital, Sydney Local Health District, Camperdown, New South Wales, Australia; 7 Wiser Healthcare, School of Public Health, The University of Sydney, Sydney, New South Wales, Australia; 8 The George Institute for Global Health, Faculty of Medicine and Health, UNSW Sydney, Sydney, New South Wales, Australia

**Keywords:** Randomised Controlled Trial, Emergency Departments, Musculoskeletal disorders, Health Services

## Abstract

**Introduction:**

Patients with musculoskeletal conditions often seek care in an emergency department (ED). The problem is that the time required to manage these patients places an additional pressure on ED physician and nursing staff, who are primarily trained and resourced to manage high-acuity patients. Primary-contact physiotherapists could play a greater role in supporting ED physician and nursing staff in the management of patients presenting to the ED with musculoskeletal conditions.

**Methods and analysis:**

The RESHAP-ED trial is a multicentre, pragmatic, open-label, two-arm, parallel randomised controlled trial with nested process and economic evaluations. The trial will investigate whether a primary-contact physiotherapy pathway compared with usual care (primary-contact by physician and/or nursing staff pathway) reduces time spent in ED. Patients with simple musculoskeletal conditions will be recruited from EDs in New South Wales, Australia. The primary outcome is ED length of stay (LOS). Secondary outcomes will include acceptability, feasibility and cost-effectiveness of primary-contact physiotherapy, and explore patients’ and clinicians’ experience. To detect a 30 min between-group difference in ED LOS, 1370 patients will be required. Analyses of the primary and secondary outcomes will be conducted following the intention-to-treat principle. The adjusted mean difference in ED LOS and 95% CI will be calculated using linear regression adjusted for hospital using a random effect model.

**Ethics and dissemination:**

The study received ethical approval from the Sydney Local Health District (RPAH zone) Human Research Ethics Committee (X23-0143). Findings from this study will be disseminated through publication in peer-reviewed journals and conference presentations.

**Trial registration number:**

Australia New Zealand Clinical Trials Registry: ACTRN 12623000782639; Universal Trial Number (UTN): U1111-1292-2883.

**STRENGTHS AND LIMITATIONS OF THIS STUDY**

- As a pragmatic trial, the intervention will be carried out in real-world emergency department (ED) settings, and the results can be immediately applied in routine practice.
- This is a multicentre trial, including metropolitan and regional EDs, optimising patient recruitment and generalisability of the results.
- This trial includes nested economic and qualitative evaluations to investigate the cost-effectiveness and acceptability of primary-contact physiotherapy in EDs.
- As an open-label trial, clinicians and patients will not be blinded to treatment allocation, but trial investigators and statisticians will remain blinded to reduce bias.

## Introduction

### Background and rationale

 Musculoskeletal conditions are common in emergency departments (EDs). In 2022–2023, there were over 432 900 visits for care of a musculoskeletal condition to EDs in Australia.^1^ The time required for musculoskeletal care and discharge planning in the ED places an additional pressure on physician and nursing staff who are primarily trained and resourced to manage high-acuity patients.

‘Primary-contact clinician’ refers to the first healthcare professional who assesses patients in the ED. In EDs, these are usually physician and/or nurse practitioners, even though in the community setting primary-contact care is often delivered by general practitioners (GPs) and allied health professionals, such as physiotherapists, for some of the conditions that people occasionally present with in the ED. Physiotherapists could play a greater role in the initial assessment and management of patients presenting to ED with a musculoskeletal condition. In addition, management and discharge planning, especially for older patients with comorbidities, is time consuming when done well, but can be delegated to an ED primary-contact physiotherapist, who is trained to work within an advanced practice physiotherapy model of care.

Preliminary evidence suggests that timely access to physiotherapy in EDs reduces waiting time, overall ED length of stay (LOS) and is well received by physician and nurse staff.^2 3^ For instance, evidence from observational data suggests that a primary-contact physiotherapy service in EDs may reduce patient waiting time by half and may reduce ED LOS by approximately 30–80 min compared with patients seeing other healthcare providers.^4^,^6^ Relying solely on physician and nurse practitioners can result in people with musculoskeletal conditions waiting longer in EDs as these clinicians have to prioritise more urgent patients. A systematic review recently published by our group reported that there are only five randomised trials in this area, and none has measured the impact of primary-contact physiotherapy on ED patient flow or ED LOS.^7^ Currently, there is one ongoing cluster randomised controlled trial conducted in Canada comparing the effectiveness of an advanced practice physiotherapist-led model of care with usual ED physician care.^8^ Although primary-contact physiotherapists have been working in Australian EDs for approximately 20 years and are considered a standard care pathway in many hospitals, there is a great deal of variation in physiotherapist involvement in the ED setting.^9^,^11^ This may be because high-level evidence of clinical and cost-effectiveness of primary-contact physiotherapy care in EDs is lacking.^12 13^

We are proposing a randomised trial to compare two approaches, both standard of care in Australia, to the assessment and management of patients who present to ED with simple musculoskeletal conditions. The more common care pathway (control group in this study) is that patients are first seen by a physician and/or nurse practitioner for assessment and management and may subsequently be referred to a physiotherapist. A less common, but well-established care pathway (intervention group in this study) involves initial assessment and management by a primary-contact physiotherapist.

### Hypothesis

Our hypothesis is that a primary-contact physiotherapy pathway in the ED to primarily assess and manage patients presenting with musculoskeletal conditions will reduce ED LOS, provide comparable health service outcomes and satisfaction with care, and be cost-effective and acceptable to patients and clinicians.

### Objectives

The RESHAP-ED trial aims to determine the clinical effectiveness, cost-effectiveness and acceptability of a primary-contact physiotherapy pathway compared with usual care (where patients are primarily managed by physician and/or nurse practitioners) for patients with musculoskeletal conditions presenting to EDs.

### Primary objective

The primary objective of this trial is to determine if a primary-contact physiotherapy pathway reduces ED LOS. The primary objective of the RESHAP-ED trial addresses the important issue of patient flow in EDs.

### Secondary objectives

The secondary objectives of the trial are to (1) investigate key health service outcomes (eg, duration of clinical care, re-presentation rates), (2) explore patient-reported outcomes (ie, pain, quality of life, satisfaction with care, adverse events), (3) determine cost-effectiveness and (4) conduct process evaluation to understand the acceptability and feasibility of the primary-contact physiotherapy pathway in ED.

## Methods and analysis

### Study design and registration

The RESHAP-ED trial is a pragmatic, multicentre, open-label, two-arm, parallel randomised controlled trial with nested process and economic evaluations. The trial protocol has been reported according to the Standard Protocol Items: Recommendations for Interventional Trials statement guidelines.^14^ The completed randomised trial and its results will be reported according to the Consolidated Standards of Reporting Trials and the Consolidated Health Economic Evaluation Reporting Standards 2022 statements.^15 16^ This study received ethical approval from the Sydney Local health District (SLHD) HREC (RPAH zone, reference number X21-0143, 13 June 2023), and the trial was prospectively registered with the Australian and New Zealand Clinical Trial Registry (ANZCTR: 12623000782639).

### Study setting

The trial will be conducted in the EDs of three metropolitan hospitals (Royal Prince Alfred, Sutherland and Ryde Hospitals), and two regional hospitals (Gosford and Wyong Hospitals) in New South Wales, Australia.

### Participants

All consecutive patients who present with a musculoskeletal condition to the participating EDs during the time the primary-contact physiotherapists are on shift will be screened by the physiotherapists for eligibility.

To be included in the trial, patients must fulfil all the following inclusion criteria: (1) adult patient 18 years or older; (2) present with a simple musculoskeletal condition. This would include, but is not limited to, soft tissue injuries (joint, ligament, tendon or muscle pain), musculoskeletal neck and low back pain, closed peripheral fractures not requiring reduction or orthopaedic fixation (indicated from triage notes), spontaneously reduced joint dislocations; and (3) have a triage score of either 3 (urgent), 4 (semi-urgent) or 5 (non-urgent) as per the Australasian Triage Scale (ATS). Patients with a triage score of 2 will also be included when presenting with an isolated joint dislocation, including shoulder, digit, patella or temporomandibular joint (indicated by triage notes).^17^

Patients will be excluded if presenting with at least one of the following exclusion criteria: (1) already assessed by a physician and/or a nurse practitioner in the ED as indicated on the electronic Medical Record (eMR); (2) any non-musculoskeletal/non-orthopaedic condition, such as open wounds, eye problems, foreign bodies and poisonings; (3) presentations outside the primary-contact physiotherapists’ working hours; (4) the triage notes indicate soft tissue injuries that may require surgical intervention; (5) the triage notes indicate a fracture requiring reduction and/or surgical fixation; (6) the triage notes indicate signs or symptoms of serious pathology (eg, spinal fracture, cauda equina syndrome, malignancy, infection or inflammatory origin of pain); (7) comorbidities requiring immediate emergency care (eg, cardiac, respiratory; or psychiatric issues as indicated on the triage note); (8) patient is unable to provide sufficient informed consent to participate (eg, cognitive impairment, intoxication, unable to understand spoken or written English) and (9) re-presentation of a patient who has already been included in the trial.

### Patient selection and recruitment

**Figure 1:**
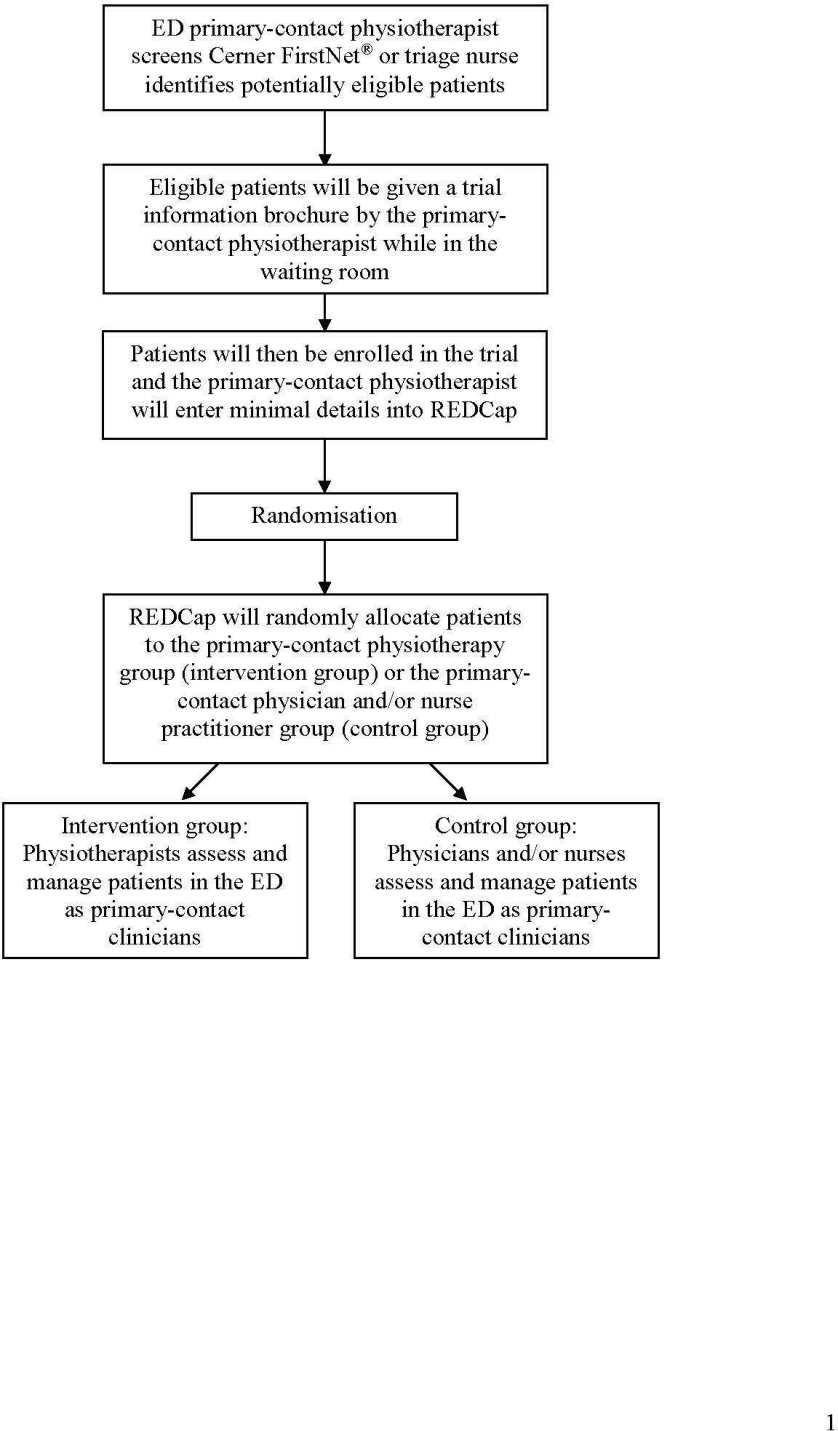
Flow of participants through the study. ED, emergency department; REDCap, Research Electronic Data Capture.

Every person presenting to the ED with a musculoskeletal condition will be triaged by nursing staff and be recorded in the ED’s electronic patient list (Cerner FirstNet^®^). This triage process will not constitute a formal clinical assessment by a healthcare provider, such as a nurse practitioner. Throughout their shift, the physiotherapists will review the ED’s electronic patient list to identify potentially eligible patients. This is a normal practice for physiotherapists within EDs in Australia. For each patient where the visit reason in the triage notes suggests a musculoskeletal condition appropriate for primary-contact physiotherapy management, the physiotherapist will fill out an electronic eligibility form in the Research Electronic Data Capture (REDCap) database. If the patient is found to be eligible during the initial screening, the physiotherapist will inform the patient about the trial and provide the trial information flyer. A waiver of consent for screening and randomisation was granted by the Ethics Committee as this study is testing the effectiveness of two standard of care pathways on ED patient flow. Flow of participants is presented in figure 1.

### Randomisation

All patients who fulfil the eligibility criteria will be randomised into the trial by the physiotherapist using REDCap. A 1:1 randomisation allocation sequence, incorporating randomly permuted block sizes of 2 and 4, and stratification by hospital site, will be generated prior to the commencement of the trial by an independent statistician not involved in participant recruitment, treatment, follow-up or statistical analysis using Statistical Analysis System (SAS) software. The randomisation schedule will be uploaded into REDCap to allow the randomisation to be performed online by the physiotherapist. Patients will be randomly allocated to either the primary-contact physiotherapy group (intervention group) or to the medical and/or nurse practitioner group (control group). REDCap will allocate each patient a participant ID number.

### Blinding

Due to the nature of the intervention, complete blinding will not be possible. Patients and clinicians will not be blinded to group allocation. The researcher downloading the eMR data, including primary outcome, will be blinded to group allocation. The statistician analysing the data and the investigators interpreting the results will also be blinded. The group allocation will be revealed to all investigators once the analyses are completed, and results are validated using randomly scrambled group allocations.

### Trial clinicians

Trial clinicians included in the RESHAP-ED trial will be ED primary-contact clinical staff, such as physicians, nurses and physiotherapists, who routinely manage patients presenting to EDs with a primary complaint of a musculoskeletal condition. For each ED site, at least one primary-contact physiotherapist (level 4) will be employed to be responsible for the patient enrolment process and the primary-contact physiotherapy pathway assessment and management. The research team will be responsible for the training of all ED staff working in the trial to make sure the trial procedures are followed as per study protocol and the Australian Code for the Responsible Conduct of Research, 2018.^18^

### Trial intervention and control

#### Primary-contact physiotherapy group (intervention group)

Following randomisation, patients who are allocated to the primary-contact physiotherapy group will be flagged in the eMR system. Recording who provides initial care in the system is a standard procedure in Australian EDs.

The physiotherapy intervention will be delivered by a senior physiotherapist (level 4 in Australia), a highly experienced clinician responsible for advanced patient assessment and management. As primary-contact clinician, they can independently diagnose and treat patients. In Australian EDs, a primary-contact physiotherapist is authorised to refer patients for diagnostic imaging and specialist consultations, with some jurisdictions also permitting them to prescribe a limited range of medications. Following an initial assessment, the primary-contact physiotherapist will choose the best course of management in consultation with the patient. In this trial, management of patients presenting with symptoms of low back pain will be provided following recommendations from the Australian Low Back Pain Clinical Care Standard.^19^ For any other musculoskeletal condition presentation, the clinical care will be guided by the best practice advice recommended by the NSW Emergency Care Institute.^20^

The primary-contact physiotherapist may consult an ED physician and/or nurse practitioner (collaborative model of care) or autonomously manage the clinical care of patients. In the autonomous model of care, the physiotherapist is the primary-contact clinician responsible for the patient assessment, management, diagnosis and discharge with no involvement of a physician and/or nurse practitioner. In the collaborative model of care, after selecting and assessing the patient, the primary-contact physiotherapist shares management and discharge decision-making with a physician and/or nurse practitioner.

In this trial, all patients will be provided with a discharge plan, including timely follow-up in the outpatient setting. Outpatient care will be provided by existing outpatient services such as outpatient physiotherapy, virtual hospitals, GP clinics, or specialist clinics, if required.

#### Primary-contact physician and/or nurse practitioner group (control group)

The control group will consist of primary-contact clinical care delivered by an ED physician and/or nurse practitioner in line with normal practice at the site. Patients allocated to the control group will not be seen by the primary-contact physiotherapist and will wait to be seen by a primary-contact physician and/or nurse practitioner. Physician and/or nurse practitioners may refer patients to see a physiotherapist in the ED as secondary-contact or may refer patients to the outpatient physiotherapy department following discharge (collaborative model of care).

### Data collection and study follow-ups

#### Data collection procedures

Health service outcomes and patient demographic characteristics will be extracted directly from the hospital eMR. Patient-reported outcomes will be collected via an online survey, supplemented by telephone calls. At days 1, 7 and 42 following randomisation, participants will receive a text message including a link for the e-Consent Form and online survey. One reminder message will be sent to non-responders at each follow-up time point. If no response is received, a trial research officer will contact them via telephone to complete the assessment.

#### Data management and monitoring

Research data from online surveys will be stored into REDCap; data from hospital eMR will be stored in password-protected folders on the University of Sydney server. A master code sheet will enable the data extracted directly from the hospital eMR to be reidentified via the patients’ medical record number to allow for data validation and audit as required, but only available to the trial coordinating principal investigator. The data will be scrutinised for omissions and errors and will be archived on the University of Sydney server for 15 years, after which data will be deleted.

### Outcome measures

#### Primary outcome

The primary outcome for this trial will be ED LOS; defined as the period between when a patient presents at an ED, and when that patient is discharged from the ED.

#### Secondary outcomes

##### Health service outcomes

- Time to clinical care; defined as time elapsed in minutes for each patient from ED presentation to the commencement of ED (non-admitted) clinical care.
- Duration of clinical care; defined as the period between when clinical care commences and the end of the (non-admitted) patient ED clinical care.
- Proportion of patients whose ED LOS is ≤4 hours (ie, proportion with care completed within 4 hours).
- Proportion of patients ‘seen on time’; defined as the proportion of presentations for which the waiting time to commencement of clinical care was within the benchmark time specified in the definition of the ATS category.^21^
- Proportion of patients admitted to the ED Short Stay Unit.
- Proportion of patients admitted to hospital inpatient units.
- Proportion of patients who received imaging.
- Proportion of patients who received opioids.
- Proportion of patients who received gabapentinoids.
- Proportion of patients who received specialist consult within the ED.
- Proportion of patients referred to specialist clinic.
- Proportion of patients re-presenting to the ED for the same chief complaint within 72 hours of discharge.
- Proportion of patients readmitted to the hospital inpatient units for the same chief complaint within 30 days of index inpatient admission.

##### Patient-report outcomes

- Average pain intensity measured using the numeric rating scale ranging from 0 (no pain) to 10 (worst imaginable pain). This outcome will be measured at days 1, 7 and 42 following ED discharge.
- Quality of life measured using the EuroQol 5-Dimension 5-Level (EQ-5D-5L).^22 23^ This outcome will be measured at days 1, 7 and 42 following ED discharge.
- Satisfaction with ED care measured using item 31 of the ED Patient Experience of Care survey advocated by the American College of Emergency Medicine.^24^ This outcome will be measured at day 1 following ED discharge.
- Adverse events will be assessed on day 1 following ED discharge. Participants will complete an online survey that will include a question prompting participants to report the occurrence of any adverse event during the ED stay. Participants experiencing an adverse event will have the opportunity to include more information about the adverse event. The investigators will review the clinical notes of participants reporting adverse events and extract adverse event data. The Medical Dictionary for Regulatory Activities will be used to code adverse event data. A blinded independent assessor will review adverse event data and assign attribution to study interventions.

The trial’s schedule of assessment is presented in table 1.

**Table 1:** Trial schedule of assessment

Procedures	Day 0 (emergency department visit)	Day 1	Day 7	Day 42
Patients screened for inclusion/exclusion criteria	✓			
Participants given trial flyer	✓			
Participants randomised via Research Electronic Data Capture	✓			
Interventions—physiotherapy or physician/nurse pathway	✓			
Participants invited by physiotherapist to qualitative interviews	✓			
SMS invite to online questionnaire and e-Consent		✓		
Online questionnaire completion—pain intensity, EuroQol 5-Dimension 5-Level		✓	✓	✓
Online questionnaire completion—satisfaction with care, adverse events		✓		
Completion of qualitative interviews	Patient’s interview—completed between day 1 and day 42 post emergency department discharge following randomisation.Clinician’s interview—completed after 50 patients are randomised at a participating site.			

### Statistical methods

#### Sample size calculation

With a total sample size of 1370 patients (685 per arm), the study will have more than 90% power to detect a difference of 30 min in ED LOS, assuming an SD of 115 min. This calculation assumes a *t*-test with equal variance in both groups. This sample size will also give us 80% power to detect a 30 min between-group difference in subgroups of soft tissue injuries and fracture/dislocation, assuming 60% and 40% of patients with soft tissue injuries and fracture/dislocation, respectively. We believe the fracture/dislocation cohort may not accurately show the advantage of the primary-contact physiotherapy pathway as the LOS may be impacted by external factors such as waiting for an orthopaedic consult.

A difference of 30 min is considered clinically important based on previous evidence suggesting that 30 min was associated with a decrease in representation and ‘left without being seen’ rates.^25^ We also had input from local opinion leaders, including local ED directors who reported 30 min was clinically important and would impact patient flow. An SD of 115 min is consistent with a recent Australian study on quality indicators for musculoskeletal injuries in the ED.^26^ We do not anticipate any missing data for the primary outcome since LOS is routinely recorded for every patient presenting to an ED.

#### Effectiveness analysis plan

The main analysis of the primary and secondary outcomes will be conducted following the intention-to-treat principle, that is, by analysing patients according to their randomised group, regardless of post-randomisation events. The main analysis of ED LOS will be performed using linear regression adjusted for hospital using a random effect model. The effect of the intervention will be estimated as the adjusted mean difference and 95% CI. While ED LOS is unlikely to follow a normal distribution, the large sample size will allow us to make inferences about the mean difference. In case of important heteroscedasticity, as assessed using a plot of residuals against fitted values, we will conduct a sensitivity analysis using a log-transformation.

A detailed analysis plan including mock tables will be developed prior to unblinding. Analyses prespecified in the analysis plan will be programmed using randomly scrambled treatment allocations. Unblinded results will be presented to the study team once all analyses have been programmed and validated and the database locked. Analyses will be performed using SAS (V.9.4 or higher) or R (V.4.3.1 or higher).

#### Economic evaluation

An economic evaluation, including cost-utility and cost-effectiveness analyses, of the primary-contact physiotherapy pathway compared with the physician and/or nurse practitioner pathway will be undertaken from the hospital perspective and according to the intention-to-treat principle. Data from the hospital on healthcare utilisation costs using presentation-level costing data will be collected. Intervention costs will be collected from trial records. Patient-level costing data will be calculated using the reported clinical and administrative activity received by each patient during their hospital visit. The method to collect intervention costs and the included costing data have been reported in our previous study.^27^ The effectiveness outcomes of the economic evaluation will be (1) quality-adjusted life-years (QALYs) gained over 6 weeks (cost-utility analysis) and (2) time spent in the ED avoided (cost-effectiveness analysis).

Utility-based quality of life will be measured using the EQ-5D-5L questionnaire,^22 23^ which has five domains with five levels each (mobility, self-care, usual activities, pain/discomfort and anxiety/depression), and the health utility score, which is expressed as an index value for health, will be calculated from the five domains for each participant in the trial using published Australian scoring algorithms.^28^ The score falls on the 0.0 (dead) to 1.0 (perfect health) value scale.

QALYs will be calculated using the health utility score calculated for the 6-week time point (day 42 post-trial randomisation).

The Incremental Cost-Effectiveness Ratios (ICERs) for each effectiveness outcome expressed as additional cost per (1) QALY gained at 6 weeks (cost-utility analysis) and (2) each additional person spending at least 30 min less in the ED (cost-effectiveness analysis) will be calculated using the mean costs and the mean health outcomes in each trial arm. Uncertainty around the ICERs will be explored using a non-parametric bootstrapping analysis (5000 replications) to generate 95% CIs that will be plotted on an incremental cost-effectiveness plane. Cost-effectiveness acceptability curves will also be estimated to show the probability that the primary-contact physiotherapy pathway is cost-effective compared with the usual care group and when considering different decision maker’s willingness to pay per QALY and for each additional person spending at least 30 min less in the ED. In Australia, there is no explicit willingness to pay threshold as it depends on the decision-making context; therefore, results will be presented using a cost-effectiveness acceptability curve, illustrating the probability of cost-effectiveness across a range of willingness to pay thresholds. Costs will be expressed in Australian dollars, adjusted for inflation using the Health Consumer Price Index as published by the Australian Bureau of Statistics. We will use multiple imputation methods to address missing data for the economic evaluation as suggested by Faria *et al.*^29^

#### Process evaluation

A series of semistructured qualitative interviews will be undertaken to assist in the trial process evaluation. About 40 ED clinicians and 40 trial participants managed by primary-contact physiotherapists will be invited to participate. Clinicians will be purposively selected to vary by profession, age, gender and experience working in the ED. Trial participants will vary in gender, age, musculoskeletal condition, and triage category.

Only patients randomised to the primary-contact physiotherapy group will be verbally invited to participate in the qualitative interviews by their treating primary-contact physiotherapist during the ED visit. If participants agree, the primary-contact physiotherapist will provide a copy of the participation information sheet and will get participants’ consent to share their preferred contact details with the research team. The research team will then contact interested participants to explain the semistructured qualitative interview in more detail and gain informed verbal consent. Participants’ semistructured qualitative interviews will occur between days 1 and 42 following study randomisation.

The ED clinicians (physician and nurse practitioners, and physiotherapists) of each involved hospital ED will also be invited to participate in the semistructured qualitative interview. Clinicians will receive an invitation email from the head of department to take part in the trial qualitative interview and will be asked to read the participant information sheet and consent form attached in the email. Clinicians who agree to participate will be invited to register their interest via a REDCap link and contacted by the research team. Clinicians' semistructured qualitative interviews will occur after 50 patients are randomised at a participating hospital ED.

The interviews will be semistructured, audio-recorded and transcribed, and will follow the study procedures. The audio recordings will be stored in a password-protected University of Sydney drive and deidentified. The main aim of the interviews will be to qualitatively explore the implementation outcomes of acceptability and feasibility by examining clinicians’ experiences, opinions, barriers and facilitators, as well as patient’s experiences and overall satisfaction with the primary-contact physiotherapy care pathway.

## Ethics and dissemination

This trial protocol is in accordance with the principles of the Declaration of Helsinki. This study received ethical approval from the SLHD HREC (RPAH zone, protocol number X21-0143), Sydney, Australia, on 13 June 2023. A waiver of consent was granted by the SLHD HREC (RPAH zone) for patients to be enrolled in this trial. The obtained results will be submitted to peer-reviewed publications and will be presented at national and international conferences.

### Patient and public involvement

We have engaged with consumers and clinician-researchers from study conception. We sought feedback from emergency clinicians of the Sydney Local Health District and the RPA Green Light Institute for Emergency Care for the MRFF grant proposal and the study protocol. A consumer with lived experience sits on the trial steering committee and actively participate in meetings to provide ongoing feedback on trial conduct, reporting and interpretation.

### Trial status

Participant recruitment started on 22 November 2023, and the study is expected to be completed by June 2025.
