# Supplementary material for: Effectiveness of primary-contact physiotherapy in managing musculoskeletal conditions in emergency departments: protocol for the RESHAP-ED randomised controlled trial
Source: BMJ Open. 2025 Mar 12;15(3):e096044. doi: 10.1136/bmjopen-2024-096044 (PMC11904353; doi:10.1136/bmjopen-2024-096044)
Supplement: Supplementary file 1 [file bmjopen-2024-096044.reviewer_comments.pdf]

PEER REVIEW HISTORY

BMJ Open publishes all reviews undertaken for accepted manuscripts. Reviewers are asked to complete a checklist review form and are provided with free text boxes to elaborate on their assessment. These free text comments are reproduced below.

ARTICLE DETAILS

Title (Provisional)

Effectiveness of primary-contact physiotherapy in managing musculoskeletal conditions in emergency departments: protocol for the RESHAP-ED randomised controlled trial

Authors

de Campos, Tarcisio Folly; Coombs, Danielle; Sigera, Chathurani; Williams, Christopher; Rogan, Eileen; Edwards, James; McCaffery, Kirsten; Howard, Kirsten; Billot, Laurent; Maher, Chris; Machado, Gustavo C; Investigators, RESHAP-ED

VERSION 1 - REVIEW

**Reviewer**	**1**
**Name**	**Lafrance, Simon**
**Affiliation**	**Université de Montréal Faculté de Médecine, School of Rehabilitation**
**Date**	**14-Jan-2025**
**COI**	**None**

This is an interesting protocol on this advanced practice physiotherapy in the ED. I do not see any flaws in the protocol but a few aspects, especially regarding the economic evaluation lack details. Here are my comments to improve the manuscript.

1. By definition, the model that you are studying is an advanced practice physiotherapy model of care. Although I would suggest renaming it advanced practice physiotherapy models, it is also ok to call it primary-contact physiotherapy and you probably already add that reflection. However, I believe that you should at least be mentioned, either in the introduction or in the methods, that it is an advanced practice physiotherapy model of care (e.g.: you can say that these models are often referred as advanced practice physiotherapy models of care in the introduction if you do not want to use that terminology).

2. P6. Lines 15-18: You mentioned, ‘This will be the first large, high-quality randomized trial investigating the (cost-)effectiveness and acceptability of primary-contact physiotherapy in EDs.’ However, this is not true as another trial is currently conducted in Canada.

3. Matifat, E., Berger Pelletier, E., Brison, R., Hébert, L. J., Roy, J. S., Woodhouse, L., ... & Desmeules, F. (2023). Advanced practice physiotherapy care in emergency departments for patients with musculoskeletal disorders: a pragmatic cluster randomized controlled trial and cost analysis. Trials, 24(1), 84.

4. P.9 Lines 42-47: I recommend that you also report the CHEERS https://www.equator-network.org/reporting-guidelines/cheers/

5. P.13 lines 12-13: You mention, 'The physiotherapy intervention will be delivered by a senior physiotherapist (level 4 in Australia)' Can you provide a bit more details for non-Australians?

6. P.13: Can you provide further details regarding the model of care? Notably, can the physiotherapists prescribe imaging tests, medications, refer directly to medical specialists?

7. P.16 Lines 3-8: You are measuring average pain intensity. If it's not too late in your procedure, I would suggest that you consider using the Brief Pain Inventory instead.

8. P.18 Lines 18-22: You mention that 'Data from the hospital on healthcare utilization costs using presentation level costing data will be collected. Intervention costs will be collected from trial records.' More details regarding your method to collect costs and the included costs are needed.

9. P.18 Lines 42-45: Please provide more details regarding how the QALYs will be calculated. Area under the curve method?

10. P.19: What would be your method to address missing data for the economic evaluation?

11. P.19 Lines 10: Please provide in advance the WTP threshold that you are planning to use.

12. P.19-20: Please provide more details regarding the content of the semi-structured interviews.

**Reviewer**	**2**
**Name**	**Newman, Meredith**
**Affiliation**	**Oxford University Hospitals NHS Foundation Trust**
**Date**	**25-Jan-2025**
**COI**	**None**

Overall, this is well structured, easy to read, the methods clearly match the study objectives and will achieve the stated objectives. For the most part, the authors present sufficient detail.

Comments:

I agree that the use of physiotherapists in ED is routine but not necessarily standardised, and I think it is worth noting in your introduction that these roles occur across the world (not only in Australia). References to a 'level 4' physiotherapist in the paper is very specific to an Australian eastern coast setting and difficult to interpret for a wider audience. For example, a level 4 is someone employed as a physiotherapy support worker in the UK. Please consider changing this and describing the skills themselves e.g. registered physiotherapist with advanced practice skills, had additional training in ED environment?

Please consider your explanation of how patients are identified by a physiotherapist in relation to the triage system. That is are the physiotherapists themselves conducting the triage? Your exclusion criteria state that patients will not be eligible if they have already been assessed by a different clinician but also that patients who have been triaged as level 3/ 4 etc will be eligible. I can guess what is happening but I think for those who do not work consistently in and Australian ED setting your explanations of inclusion/ exclusion criteria could be tighter and the process in relation to triage needs to be more explicit.

Could you please note who will be doing the phone call follow ups to collect data. I assume a research assistant?

VERSION 1 - AUTHOR RESPONSE

Reviewer #1

Mr. Simon Lafrance, Université de Montréal Faculté de Médecine, Hopital Maisonneuve-Rosemont Centre de Recherche

Comments to the Author:

This is an interesting protocol on this advanced practice physiotherapy in the ED. I do not see any flaws in the protocol but a few aspects, especially regarding the economic evaluation lack details. Here are my comments to improve the manuscript.

1. By definition, the model that you are studying is an advanced practice physiotherapy model of care. Although I would suggest renaming it advanced practice physiotherapy models, it is also ok to call it primary-contact physiotherapy (PCP) and you probably already add that reflection. However, I believe that you should at least be mentioned, either in the introduction or in the methods, that it is an advanced practice physiotherapy model of care (e.g.: you can say that these models are often referred as advanced practice physiotherapy models of care in the introduction if you do not want to use that terminology).

Response: Thanks for the suggestion. To make it clear that the PCP is trained to work within an advanced practice physiotherapy model of care; we have modified the last sentence in the second paragraph of the *Introduction* section. The new sentence now reads:

> *"In addition, management and discharge planning, especially for older patients with comorbidities, is time consuming when done well, but can be delegated to an ED primary-contact physiotherapist, which is trained to work within an advanced practice physiotherapy model of care."*

2. P6. Lines 15-18: You mentioned, 'This will be the first large, high-quality randomized trial investigating the (cost-)effectiveness and acceptability of primary-contact physiotherapy in EDs.' However, this is not true as another trial is currently conducted in Canada (Matifat, E., Berger Pelletier, E., Brison, R., Hébert, L. J., Roy, J. S., Woodhouse, L., ... & Desmeules, F. (2023). Advanced practice physiotherapy care in emergency departments for patients with musculoskeletal disorders: a pragmatic cluster randomized controlled trial and cost analysis. Trials, 24(1), 84).

Response: We thanks the reviewer to flag that a new trial is currently been conducted in Canada. Following the Editor's first comment, we have now revised the *Strengths and limitations of this study* section, point 3 on page 6. The new sentence now reads:

> "• *This trial includes nested economic and qualitative evaluations to investigate the cost-effectiveness and acceptability of primary-contact physiotherapy in EDs."*

We have also included a sentence in the Introduction section, 3rd paragraph on page 8, to inform that there is an ongoing trial in Canada comparing the effectiveness of an advanced practice physiotherapist model of care with usual ED care. We have also added a new reference (https://doi.org/10.1186/s13063-023-07100-x). The new sentence on page 9 now reads:

> *"Currently, there is one ongoing cluster randomised controlled trial conducted in Canada comparing the effectiveness of an advanced practice physiotherapist (APPT)-led model of care with usual ED physician care.[8]"*

3. P.9 Lines 42-47: I recommend that you also report the CHEERS https://www.equator-network.org/reporting-guidelines/cheers/.

Response: Thanks for the recommendation. We have modified the sentence on page 9 to add the *Consolidated Health Economic Evaluation Reporting Standards 2022 (CHEERS 2022) statement.* We have also added a new reference (https://doi.org/10.1136/bmj-2021-067975). The new sentence on page 9 now reads:

> *"The completed randomised trial and its results will be reported according to the Consolidated Standards of Reporting Trials (CONSORT), and the Consolidated Health Economic Evaluation Reporting Standards 2022 (CHEERS 2022) statements.[15 16]"*

4. P.13 lines 12-13: You mention, 'The physiotherapy intervention will be delivered by a senior physiotherapist (level 4 in Australia)' Can you provide a bit more details for non-Australians?

Response: In Australia, physiotherapists are classified into different levels based on their clinical experience, expertise, and responsibilities. A Level 4 physiotherapist is a senior clinician with advanced skills in assessment, treatment, and clinical decision-making. They typically have extensive experience in their field and may be involved in mentoring junior staff, supervising students, and contributing to service development. In the context of this study, a Level 4 physiotherapist ensures that the intervention is delivered with a high level of clinical expertise and consistency. To clarify the Physiotherapy classification level in Australia we have modify the sentence on page 13. The edited sentence now reads:

> *"The physiotherapy intervention will be delivered by a senior physiotherapist (level 4 in Australia), a highly experienced clinician responsible for advanced patient assessment and management. As primary-contact clinician, they can independently diagnose and treat patients."*

5. P.13: Can you provide further details regarding the model of care? Notably, can the physiotherapists prescribe imaging tests, medications, refer directly to medical specialists?

Response: To expand details on the scope of practice of a primary-contact physiotherapist (level 4 in Australia) we have added a new sentence on page 13. The new sentence reads:

"In Australian EDs, a primary-contact physiotherapist are authorised to refer patients for diagnostic imaging and specialist consultations, with some jurisdictions also permitting them to prescribe a limited range of medications."

6. P.16 Lines 3-8: You are measuring average pain intensity. If it's not too late in your procedure, I would suggest that you consider using the Brief Pain Inventory instead.

Response: We thank the reviewer for the suggestion; however, we will not be able to modify the study protocol to use the Brief Pain Inventory as we have already commenced participant recruitment.

7. P.18 Lines 18-22: You mention that 'Data from the hospital on healthcare utilization costs using presentation level costing data will be collected. Intervention costs will be collected from trial records.' More details regarding your method to collect costs and the included costs are needed.

Response: We have included a new sentence in the Economic evaluation section, first paragraph on page 18 to add information to direct readers to where to find details on the method to collect costs and the included costs for this trial. We have also added a new reference (https://doi.org/10.1016/j.lanwpc.2020.100089). The new sentence on page 18 now reads:

"Patient-level costing data will be calculated using the reported clinical and administrative activity received by each patient during their hospital visit, according to the NSW Health Cost Accounting Guidelines. Further details on the method to collect intervention costs and the included costing data have been reported in our previous study.[27]"

8. P.18 Lines 42-45: Please provide more details regarding how the QALYs will be calculated. Area under the curve method?

Response: See last sentence of page 18. QALYs will be calculated using the health utility score calculated for the 6-week time-point (day 42 post-trial randomisation).

9. P.19: What would be your method to address missing data for the economic evaluation?

Response: Thank you for the comment. We have included a new sentence in the *Economic evaluation* section, last paragraph on page 19 to add information on how we will address missing data for the economic evaluation. We have also added a new reference (https://doi.org/10.1007/s40273-014-0193-3). The new sentence on page 19 now reads:

> *"We will use multiple imputation methods to address missing data for the economic evaluation as suggested by Faria et al.[29]"*

10. P.19 Lines 10: Please provide in advance the Willingness to Pay (WTP) threshold that you are planning to use.

Response: We have provided a new sentence in the *Economic evaluation* section, last paragraph on page 19 to provide information on the use of willingness to pay threshold we are planning for this project. The new sentence on page 19 now reads:

"In Australia, there is no explicit willingness to pay threshold, as it depends on the decision-making context; therefore, results will be presented using a cost-effectiveness acceptability curve, illustrating the probability of cost-effectiveness across a range of willingness to pay thresholds."

11. P.19-20: Please provide more details regarding the content of the semi-structured interviews.

Response: We have now provide more details regarding the content of the semi-structured interviews. The last sentence of the last paragraph in the *Process evaluation* section on page 21 now reads:

> *"The main aim of the interviews will be to qualitatively explore the implementation outcomes of acceptability and feasibility by examining clinician's experiences, opinions, barriers, and facilitators, as well as patient's experiences and overall satisfaction with the primary-contact physiotherapy care pathway."*

Reviewer #2

Mrs. Meredith Newman, Oxford University Hospitals NHS Foundation Trust

Comments to the Author:

Overall, this is well structured, easy to read, the methods clearly match the study objectives and will achieve the stated objectives. For the most part, the authors present sufficient detail.

1. I agree that the use of physiotherapists in ED is routine but not necessarily standardised, and I think it is worth noting in your introduction that these roles occur across the world (not only in Australia). References to a 'level 4' physiotherapist in the paper is very specific to an Australian eastern coast setting and difficult to interpret for a wider audience. For example, a level 4 is someone employed as a physiotherapy support worker in the UK. Please consider changing this and describing the skills themselves e.g. registered physiotherapist with advanced practice skills, had additional training in ED environment?

Response: Please, see responses for Reviewer #1's question 4 and 5. To address this comment, we have clarified the Physiotherapy classification level and skills in Australia and we have edited the following sentences on page 13. The edited sentences now read:

> *"The physiotherapy intervention will be delivered by a senior physiotherapist (level 4 in Australia), a highly experienced clinician responsible for advanced patient assessment and management. As primary-contact clinician, they can independently diagnose and treat patients."*

> *"In Australian EDs, a primary-contact physiotherapist are authorised to refer patients for diagnostic imaging and specialist consultations, with some jurisdictions also permitting them to prescribe a limited range of medications."*

2. Please consider your explanation of how patients are identified by a physiotherapist in relation to the triage system. That is are the physiotherapists themselves conducting the triage? Your exclusion criteria state that patients will not be eligible if they have already been assessed by a different clinician but also that patients who have been triaged as level 3/4 etc will be eligible. I can guess what is happening but I think for those who do not work

consistently in and Australian ED setting your explanations of inclusion/ exclusion criteria could be tighter and the process in relation to triage needs to be more explicit.

Response: To make it clear the ED procedure in Australia and the study procedure for triaging and screening patients into the study we have edited the *Patient selection and recruitment* section on page 11. The first three sentences in this section now read:

> *"Every person presenting to the ED with a musculoskeletal condition will be triaged by nursing staff and be recorded in the ED's electronic patient list. This triage process will not constitute a formal clinical assessment by a healthcare provider, such as a nurse practitioner. Throughout their shift, the physiotherapists will review the ED's electronic patient list to identify potentially eligible patients."*

3. Could you please note who will be doing the phone call follow ups to collect data. I assume a research assistant.

Response: To make it clear who will be doing the phone call follow-ups to collect Patient-reported outcomes we have edited the *Data collection procedures* section on page 14. The last sentence in this section now reads:

> *"One reminder message will be sent to non-responders at each follow-up time-point. If no response is received, a trial research officer will contact them via telephone to complete the assessment."*
